# Personal

**Published:** 1892-07

**Authors:** 


### Personal.

---

Dr. A. J. Martin, of Clarence, N. Y., has gone abroad for a year to study in foreign universities.

---

Dr. William Easterly Ashton, formerly Lecturer on Gynecology at Jefferson Medical College, has been appointed Professor of Gynecology in the Medico-Chirurgical College, of Philadelphia, vice Montgomery, transferred to Jefferson. This is a fitting recognition of one of the brilliant members of the younger school of gynecology that has lately grown up in Philadelphia.

---

Dr. Robert Tuttle Morris, of New York, the well-known surgeon, has had conferred upon him at the recent commencement of the Center College, of Kentucky, the degree of A. M., *causa honoris.* This college is one of the oldest and most famous of the southern institutions of learning,— the Alma Mater of the Crittendens,

Breckenridges, Blackburns, Harlins, and others of national renown. The honor in case of Dr. Morris is not only worthily bestowed, but it will be secure.

---

Dr. Lewis S. Pilcher, of Brooklyn, President of the Medical Society of the State of New York, has gone abroad for a few weeks, and is expected to return sometime during August. It may be expected that Dr. Pilcher will inaugurate the campaign and prepare for the next meeting of the society very soon after his return from Europe.

---

Dr. Arch. Dixon, of Henderson, was elected President of the Kentucky State Medical Society, at its meeting recently held in Louisville. The Kentucky State Medical Society has an honorable record, is progressive in its methods, and elevates her deserving members to the chair. In the selection of Dr. Dixon for president, she has remained true to her traditions.

---

Dr. Alvin A. Hubbell has returned from his European trip. During his sojourn abroad, he visited the immense ophthalmic clinics of Moorfields, Royal Westminster Ophthalmic, Royal South London Ophthalmic, and other hospitals of London, Abadie's, Gale-Zowski's, DeWicker's, Landolt's, Quintze-Vinghts, Hôtel Dieu, and others of Paris, and everywhere he went he was most cordially received. He also enjoyed the rare hospitalities at their homes of Dr. Julius H. Mickle, Mr. Power, Mr. McHardy, and Mr. G. Anderson Critchett, of London, Mr. Priestly Smith, of Birmingham, and Dr. E. Meyer, and Dr. Landolt, of Paris.

---

Dr. T. Haven Ross has removed from his former residence, 344 Ashland avenue, to 269 Franklin street, where he will limit his practice to the treatment of diseases of the skin, including removal of hair and cutaneous growths by electrolysis.

---

Mr. William Dean Howells, editor of the *Cosmopolitan*, whose portrait appeared in the June number of the Journal, intends spending his summer in a quiet nook in New England, devoting a large portion of his time to the writing of his novel of American Girl-Life, to be published in the autumn in *The Ladies' Home Journal.*

Miss Jessamy Harte, daughter of Bret Harte, will make her literary début in the July *Ladies' Home Journal* with a most entertaining description of Camp Life in the Adirondacks, in which it is claimed every evidence shows itself of inherited literary tendencies not unlike those evidenced in her father's earlier work. Miss Harte is a girl still in her 'teens, and has artistic as well as literary proclivities, as one of the illustrations accompanying her first article shows.

Dr. Charles G. Stockton, of Buffalo, was elected chairman of the Section of Medicine, at the recent meeting of the American Medical Association, at Detroit.

